# Interactions between phasic alerting and consciousness in the fronto-striatal network

**DOI:** 10.1038/srep31868

**Published:** 2016-08-24

**Authors:** Ana B. Chica, Dimitri J. Bayle, Fabiano Botta, Paolo Bartolomeo, Pedro M. Paz-Alonso

**Affiliations:** 1 Department of Experimental Psychology, and Brain, Mind, and Behavior Research Center (CIMCYC), University of Granada, Spain; 2 Sport and Movement Research Center (CeRSM, EA 2931), Université Paris Ouest-La Défense, Nanterre, France; 3 INSERM U 1127, CNRS UMR 7225, Sorbonne Universités, and Université Pierre et Marie Curie-Paris 6, UMR S 1127, Institut du Cerveau et de la Moelle épinière (ICM), F-75013 Paris, France; 4 BCBL, Basque Center on Cognition, Brain and Language, Donostia, Spain

## Abstract

Only a small fraction of all the information reaching our senses can be the object of conscious report or voluntary action. Although some models propose that different attentional states (top-down amplification and vigilance) are necessary for conscious perception, few studies have explored how the brain activations associated with different attentional systems (such as top-down orienting and phasic alerting) lead to conscious perception of subsequent visual stimulation. The aim of the present study was to investigate the neural mechanisms associated with endogenous spatial attention and phasic alertness, and their interaction with the conscious perception of near-threshold stimuli. The only region demonstrating a neural interaction between endogenous attention and conscious perception was the thalamus, while a larger network of cortical and subcortical brain activations, typically associated with phasic alerting, was highly correlated with participants’ conscious reports. Activation of the anterior cingulate cortex, supplementary motor area, frontal eye fields, thalamus, and caudate nucleus was related to perceptual consciousness. These data suggest that not all attentional systems are equally effective in enhancing conscious perception, highlighting the importance of thalamo-cortical circuits on the interactions between alerting and consciousness.

Conscious perception depends on the activity of large-scale networks, including key nodes in the parietal and frontal cortex^1^,^2^,^3^,^4^. Beside fronto-parietal networks, thalamo-cortical circuits are also crucial for conscious perception^5^,^6^. In order to reach consciousness, three important conditions must be achieved^4^: (1) a sufficient level of bottom-up activation from early sensory regions, (2) top-down amplification produced by the reverberation of brain activation in higher association cortices^7^,^8^,^9^,^10^,^11^, and (3) a sufficient level of vigilance^12^,^13^,^14^. Although neural activation might contribute to consciousness only if it is sustained for a minimum period of time (around a few hundreds of ms), the phasic discharge of some neurons also correlates with consciousness. It has been proposed that this phasic activity might not be sufficient to produce a conscious percept in the absence of sustained firing to effectively transfer information to downstream areas^15^.

Conscious perception can also be predicted by pre-stimulus activation^16^,^17^, which is sometimes associated with spatial orienting processes before the relevant stimulus is presented^18^,^19^,^20^,^21^. In line with Dehaene and colleagues’ predictions, exogenous or bottom-up spatial attention (elicited by spatially informative peripheral cues) improves perceptual sensitivity to detect near-threshold stimuli, with increased brain activation (and functional connectivity) in fronto-parietal regions before stimulus presentation^22^. However, and contrary to Dehaene and colleagues’ proposal, top-down activation (elicited by spatially informative symbolic cues) has only a weak effect on perceptual sensitivity for near-threshold targets^22^. Accordingly, the neural correlates of endogenous spatial attention seem to be dissociable from the brain correlates of conscious processing, as reported by studies using electroencephalography (EEG)^18^ and magneto-encephalography (MEG)^21^. Our previous studies have demonstrated that, contrary to top-down or endogenous spatial attention, exogenous spatial attention interacts with conscious perception^19^,^22^, as assessed by functional interactions within specific fronto-parietal networks^20^. Finally, regarding the level of vigilance, behavioral improvements on conscious perception have been reported when the alerting system has been manipulated before the relevant target is presented. Phasic alertness temporarily improves consciousness deficits in neglect patients, while tonic alertness (sustained attention) has a positive impact in patients’ spatial biases in rehabilitation programs^23^,^24^. Phasic alertness induced by a short auditory tone also improves the conscious perception of near-threshold stimuli in healthy participants^25^,^26^.

The interactions between endogenous spatial attention and the conscious perception of near-threshold targets have been previously explored using EEG^18^ and MEG^21^,^27^, both techniques with a high temporal resolution but low spatial resolution. However, the interaction with phasic alerting has not been explored so far with neuroimaging or neurophysiological techniques. Here, we used event-related functional magnetic resonance imaging (fMRI) to investigate the brain mechanisms associated with different attentional states (endogenous orienting and alerting attentional systems) and their relation with conscious perception. We manipulated top-down amplification produced by endogenous spatial attention and phasic alertness, and explored conscious reports of near-threshold stimuli. We orthogonally manipulated both endogenous spatial attention and phasic alertness before the to-be-detected target was presented^26^, and measured their impact on the modulation of conscious perception of a near-threshold Gabor stimulus (titrated to be consciously perceived on ~50% of the trials). If both top-down amplification (endogenous spatial attention) and phasic alertness were important pre-requisites of conscious perception, then brain activations associated with both attentional mechanisms should be related to subsequent conscious reports. Based on previous behavioral evidence^22^, we predicted that brain activations associated with endogenous spatial attention within the dorsal fronto-parietal network (including bilateral brain regions of the inferior and superior parietal lobe -IPL and SPL, and frontal eye fields –FEF)^28^ would not correlate with increased probability of conscious reports. But we expected to observe, for the first time, that fronto-parietal and thalamo-cortical regions, typically involved in phasic alertness, are associated with participants’ conscious reports^26^. These regions include bilateral IPL and SPL, anterior cingulate cortex (ACC), and supplementary motor area (SMA) in the frontal lobe, and subcortical regions including caudate and thalamus^12^,^29^,^30^,^31^,^32^,^33^,^34^,^35^. We also expected that interactions between conscious perception and phasic alertness would be associated with increased functional coupling between fronto-parietal and thalamo-cortical regions^20^,^30^,^34^. According to recent proposals stating the need to disentangle the neural correlates of consciousness from its pre-requisites and consequences^2^,^3^, and given that regional and functional connectivity analyses of the present work are restricted to the cue-period (before the target is presented), our research will help to shed further light on the neural pre-requisites of consciousness.

## Results

**Figure 1:**
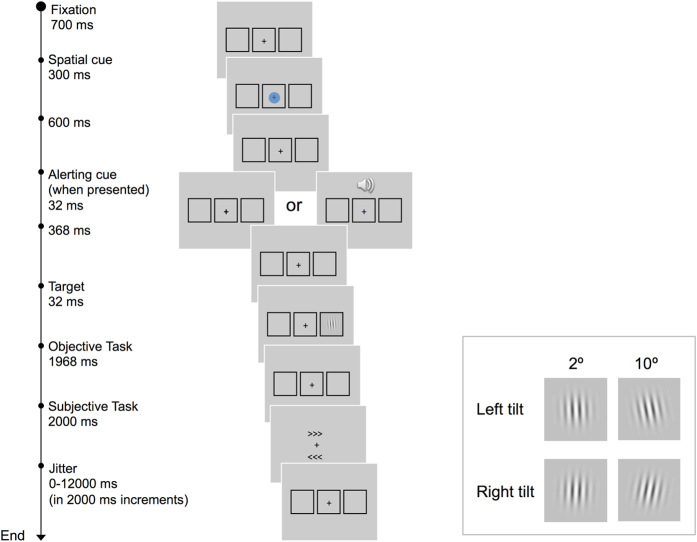
Sequence and timing of events in a given trial. The bottom-right panel shows an example of left and right tilted Gabors with the minimum (2°) and maximum (10°) tilt used in the study.

Each experimental trial started with an endogenous spatial cue (see Fig. 1). The color of this cue indicated with high probability (70%) the location of the Gabor present trials. On 13% of the trials the Gabor was absent (catch trials). The alerting cue (sound) was presented on half of the trials, in order to manipulate phasic alerting. Participants performed two consecutive responses to the Gabor: (1) an objective response (tilt orientation), used to record reaction time (RT) for both seen and unseen targets; (2) an awareness response, in which participants reported the Gabor location when it was consciously perceived (by pressing a key associated to one of the two arrows shown in the screen), or pressed the space bar when they did not see the Gabor. This paradigm allowed us to orthogonally manipulate endogenous orienting (colored cue), alerting (presence or absence of the sound), and conscious perception (Gabors were calibrated before the experimental trials -see Method section-, adjusting contrast until participants perceived ~50% of the Gabors for each condition of endogenous validity and alerting state).

### Behavioral results

As expected when using near-threshold stimuli, accuracy to discriminate tilt orientation was significantly larger for seen targets (76%) as compared to unseen targets (46%), F(1, 18) = 281.46, p < 0.001. RTs faster than 150 ms were eliminated from the RT analyses (0.053% and 0.041% of the trials for sessions I and II, respectively). Anticipatory responses (0.007% and 0.005% of the trials for sessions I and II, respectively) were also excluded. False alarms (e.g., target-absent trials in which participants reported having seen the target) accounted for 13% of the target absent trials (SD = 26.20), and were not analyzed. Errors in localizing a consciously seen target were also very rare and not further analyzed (M = 9.11, SD = 13.68).

**Figure 2:**
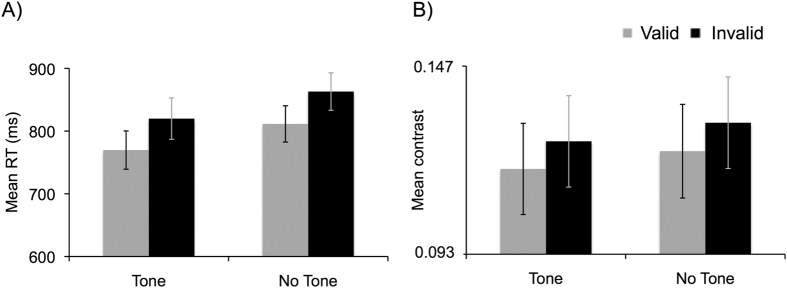
(**A**) Mean RT in the objective task for consciously reported Gabors as a function of Alerting and Validity. (**B**) Mean Michelson contrast values used to achieve ~50% “seen” and “unseen” targets (in the subjective task) as a function of Alerting State and Validity. Error bars represent standard errors.

We analyzed the RT of the objective task (reporting tilt orientation of seen targets) and target contrast values as a function of validity and alerting state by performing two repeated measures analysis of variance (ANOVA) with the factors of Session (I and II), Awareness (‘seen’, ‘unseen’), Validity (valid, invalid), and Alerting State (tone present, tone absent). Accuracy on the objective task was also analyzed with an identical ANOVA. Data from two participants were excluded from these analyses due to technical problems collecting objective task responses in one of the sessions. These two participants were included in the fMRI analysis because their responses to the subjective task were correctly recorded. Even thought target contrast was titrated to achieve ~50% “seen” and “unseen” targets in each condition, we analyzed the percentage of seen targets to ensure that the titration procedure was effective, with statistically similar results for endogenously valid and invalid trials, and for tone-present and tone-absent trials (all ps ≥ 0.146, for main effects and interactions). Mean RT data from the objective task showed the significant main effects of Session, F(1,17) = 9.71, MSE = 7461, η_p_^2^(partial eta-squared) = 0.36, p = 0.006; Validity, F(1,17) = 13.75, MSE = 6775, η_p_^2^ = 0.45, p = 0.002; and Alerting State, F(1,17) = 12.16, MSE = 5331, η_p_^2^ = 0.42, p = 0.003 (see Fig. 2). RTs were faster in Session II as compared to Session I, demonstrating a practice effect. Participants responded 50 ms faster for valid trials as compared to invalid trials, indicating that they were endogenously orienting attention to the location signaled by the endogenous cue. RTs were also 42 ms faster when the alerting tone was present as compared to tone absent conditions, demonstrating that participants perceived the alerting tone. The analysis of the mean accuracy data for the objective task revealed that none of the main effects or interactions were statistically significant (all ps ≥ 0.144).

We then analyzed the mean Gabor contrast used for each condition, to assess whether endogenous attention and alerting modulated participants’ conscious perception of the Gabor. We observed statistically significant main effects of Validity, F(1,17) = 5.78, MSE = 4.98, η_p_^2^ = 0.253, p = 0.028, and Alerting State, F(1,17) = 28.24, MSE = 0.43, η_p_^2^ = 0.62, p < 0.001. Consistent with previous studies^18^, Gabor contrast to perceive ~50% of the Gabors resulted to be lower when endogenous attention was focused on the valid as compared to the invalid location, and it was also lower for tone present as compared to tone absent conditions (see Fig. 2). None of the other main effects or interactions were statistically significant (all ps ≥ 0.383).

### fMRI results

**Figure 3:**
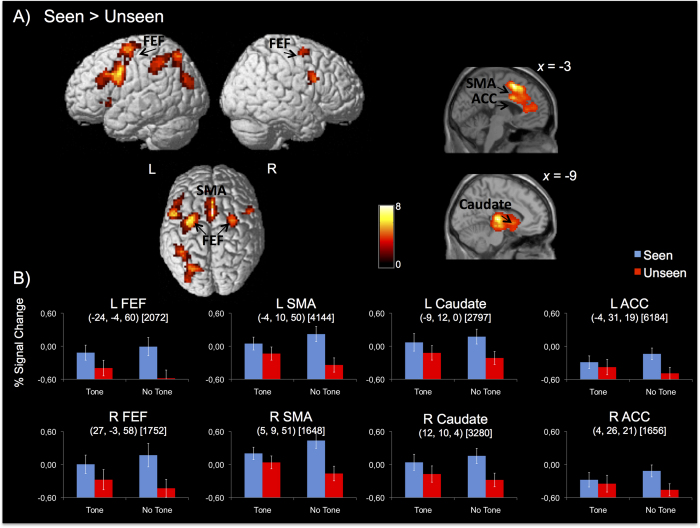
(**A**) Brain renderings showing activations for the Seen > Unseen whole-brain contrast (p < 0.05, FWE cluster-level corrected). (**B**) Percent (%) signal change from functionally identified ROIs as a function of Awareness, Alerting State, and Validity, showing a significant Alerting State x Awareness interaction (Table 2). All regions demonstrated increased % signal change for seen as compared to unseen reports, which was enhanced when the alerting tone was not presented. The center of mass of each ROI is indicated in parenthesis, and the volume in mm is indicated in squared brackets. The following ROIs were also analyzed and demonstrated no significant effects: Left IPL (−38, −48, 45) [6152], left SPL (−24, −67, 51) [3408], left thalamus [(−11, −18, 6) [7792] and right thalamus (11, −16, 7) [5032]].

A whole-brain contrast (p < 0.05, Familywise wise -FWE cluster-level corrected, voxel-level inclusion threshold *p* < 0.001) between ‘Seen’ versus ‘Unseen’ conditions revealed the regions that demonstrated larger BOLD activation during the attentional period when targets were subsequently consciously reported as compared to unreported targets (Table 1 and Fig. 3). Increased activations for this contrast were found in left IPL, extended to the left SPL, and bilateral angular gyrus (ANG). Frontal activations were observed bilaterally in the FEFs, middle frontal gyri, and insula, extending to left inferior frontal gyrus (IFG). Increased Seen > Unseen BOLD activation was also observed in the anterior and middle cingulate, and SMA, as well as bilaterally in the middle occipital lobe, and left inferior temporal lobe.

ROI analyses were conducted for the areas showing a significant modulation in the prior contrast, which have been previously related to attentional orienting or alerting (see *Methods* section). The examination of the profiles of activation within these ROIs allowed us to test for specific hypotheses about the involvement of these regions in endogenous spatial orienting and alerting for conscious and unconscious reports. For each region, we performed repeated-measures ANOVAs with Hemisphere (left and right), Validity (valid, invalid), Alerting State (tone present, tone absent) and Awareness (‘seen’, ‘unseen’ reports) as factors. For the analysis of the IPL and SPL, only the left hemisphere was examined, because the homologous right hemisphere regions were not engaged in the Seen > Unseen whole-brain contrast. We used simple-effect analyses to compare fMRI parameter estimates of each validity and alerting condition, for targets reported as ‘seen’ or ‘unseen’. These analyses revealed a group of regions showing a statistically significant Alerting State x Awareness interaction, including bilateral ACC, caudate, FEF, and SMA (see Table 2 and Fig. 3B). The interaction between Alerting State, Awareness, and Hemisphere was far from significance for all regions (all Fs < 1). As it can be observed in Fig. 3, BOLD activation was larger for seen as compared to unseen reports in all the above-mentioned regions. The effect was larger in no tone trials as compared to tone present trials.

Additional explorations of occipito-temporal ROIs derived from the Seen > Unseen contrast did not demonstrate any significant interactions implicating endogenous spatial attention, alerting, and awareness.

**Figure 4:**
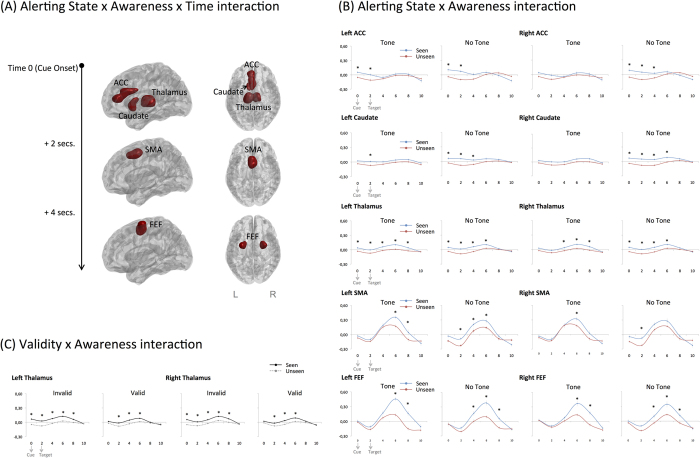
(**A**) Representation of the brain regions demonstrating an Alerting State x Awareness x Time interaction. All these regions demonstrated larger signal intensity for seen targets than for unseen targets when no alerting tone was presented. The figure represents the time interval where the differences in signal intensity started to be significant in each region, with cue onset at time 0. (**B)** Averaged signal intensity time courses for the regions demonstrating a significant Alerting State x Awareness interaction. **(C)** Averaged signal intensity time courses of the region demonstrating a significant Validity x Awareness interaction (left and right Thalamus). Time 0 represents the moment of cue onset; the target was presented 2 s (an MR frame) later. Asterisks represent significantly larger signal intensity for seen versus unseen targets at different time points (*p* < 0.05, Bonferroni corrected).

Time-course analyses also revealed Alerting State x Awareness interactions in bilateral caudate, thalamus, FEF, SMA, and ACC (see Table 3). All these regions demonstrated larger activation for seen as compared to unseen targets when no alerting tone was presented. The bilateral caudate, thalamus, and ACC presented larger signal intensity for seen than unseen trials from the moment of cue onset (all comparisons Bonferroni corrected). The effect appeared 2 seconds later in the bilateral SMA, and 2 seconds later in the bilateral FEF (Fig. 4A,B).

The thalamus was the only region demonstrating a Validity x Awareness interaction (F = 5.945, MSE = 0.019, p = 0.027, η_p_^2^ = 0.271), with an overall increased signal intensity for seen as compared to unseen targets on invalid trials but not on valid trials (Fig. 4C).

**Figure 5:**
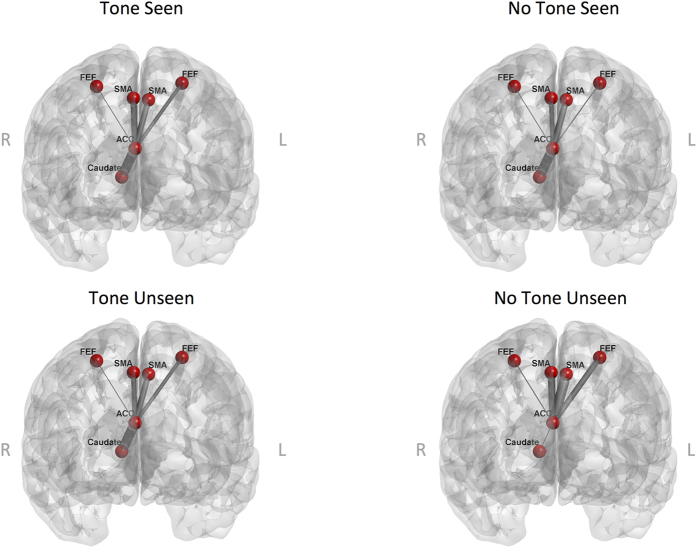
Pairwise functional connectivity for regions demonstrating a significant interaction between Alerting State and Awareness in the ANOVA of the beta-correlation values. Line thickness represents the coupling strength among regions.

To further examine interactions between spatial attention, alerting, and conscious perception networks, we conducted pairwise functional connectivity analyses between the functional ROIs. We extracted the average beta-correlation strength values for each possible pair of ROIs in each participant. These values were submitted to two separate repeated-measures ANOVAs with the factors of Validity, Alerting State, and Awareness. The right ACC area showed a significant interaction between Alerting State and Awareness for coactivations with right caudate and bilateral FEF and SMA (Table 4). All regions demonstrated similar functional connectivity for seen and unseen trials when the alerting tone was presented (see Fig. 5). However, when no alerting tone was presented, increased functional connectivity between ACC and caudate was associated with seen reports, while unseen reports were associated with reduced ACC-caudate connectivity, but increased connectivity between ACC and the SMA and FEF.

**Figure 6:**
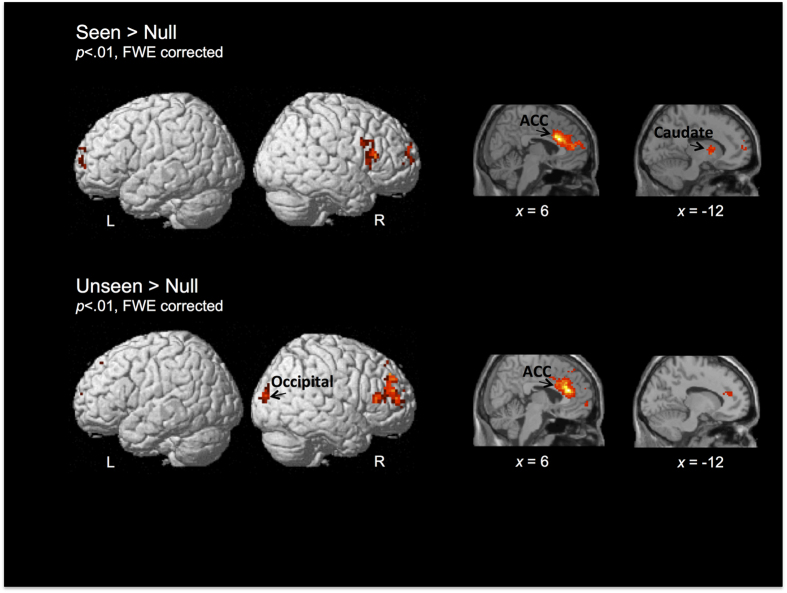
Brain renderings showing whole-brain functional connectivity analysis using the right ACC as a seed for Seen > Null and Unseen > Null contrasts (p < 0.01, FWE voxel-level corrected).

To further examine this result, we performed a whole-brain functional connectivity analysis, with a seed placed in the right ACC. This analysis showed strong functional coupling between the right ACC and bilateral inferior frontal, right middle frontal, and left caudate nucleus for seen reports (Seen > Null, p < 0.01, FWE voxel-level corrected). However, for unseen reports (Unseen > Null, p < 0.01, FWE voxel-level corrected), functional coupling was observed between the right ACC and right inferior and middle frontal gyri, left postcentral gyrus and occipital cortex (see Fig. 6). Therefore, coactivation of the right ACC with the left caudate, and the left inferior frontal region, was associated with conscious perception, while “unseen” reports were associated with coactivations of the right ACC with occipital cortex.

## Discussion

The present study was designed to examine the neural mechanisms associated with different attentional states and their relation to conscious perception. According to the Global Neuronal Workspace model^4^, both top-down amplification and alerting are necessary pre-requisites for stimuli to reach consciousness^14^. We used a paradigm in which both systems were orthogonally manipulated to explore, for the first time, their interaction with conscious perception within a single experimental paradigm. We presented participants with near-threshold Gabor stimuli while manipulating endogenous spatial attention (top-down attention) and phasic alerting. We measured behavioral indexes of conscious perception and brain activations related to ‘seen’ and ‘unseen’ reports. The behavioral results demonstrated that participants were paying attention to the location indicated by the endogenous cue, because mean RTs were shorter to respond to the attended location as compared to the unattended location. The mean target contrast necessary to consciously detect ~50% of the targets was also lower for attended as compared to non-attended spatial positions. Consistent with previous results, the alerting cue was also effective because mean RTs to respond to the target were shorter when the alerting tone was presented as compared with tone-absent conditions. Finally, mean target contrast to consciously detect ~50% of the targets was also lower with tone than without it, confirming previous results^26^. The alerting cue used in the present experiment provided more than an accessory stimulation. It provided information on the time point of target occurrence, enabling (exogenous) temporal preparation^25^,^36^,^37^. However, although the alerting effect reported in the present manuscript cannot be dissociated from temporal preparation effects, the time interval between the endogenous central cue and the target was also constant, probably reducing the impact of temporal preparation.

A large number of brain regions associated with phasic alerting were differentially engaged for seen and unseen reports. Previous studies have demonstrated that the midbrain-thalamic-anterior cingulate cortex is related to sustained^12^,^32^,^33^,^38^ and phasic alertness^29^,^31^. In our study, the ACC, caudate, SMA, and FEFs presented larger BOLD responses for seen than for unseen reports, especially when the alerting tone was absent. Our results suggest that when no alerting tone was presented, stimuli were more likely to be detected if alerting mechanisms were endogenously activated during the cue-period. A failure to activate these alerting-related regions was associated to unseen reports. In the present study we only manipulated phasic alerting, although in future studies it would be interesting to explore the effects of different levels of sustained attention in the perception of near-threshold stimuli, and to explore (and compare) the brain networks associated to sustained and phasic alerting in the modulation of conscious perception.

According to Sturm and Willmes^33^, the ACC, midbrain, and thalamus constitute the anterior alerting system, while the pre-SMA is involved in response selection and preparation^31^. The pre-SMA has also been highlighted in perceptual decision-making studies, demonstrating larger SMA activations for difficult than easy tasks^39^. In our experiments, the presence of the tone might have facilitated perceptual decision making by improving the accumulation of information, and enabling the execution of an action associated with that specific decision^40^. Our time-course analyses indicated differential activations for seen and unseen trials in the bilateral caudate, thalamus, and the ACC from the moment of cue onset, while the effect appeared 2 seconds later in the SMA. This is consistent with the Sturm and Willmes’s model, indicating that in our task, activation of the thalamus-ACC circuit preceded pre-SMA activation, and improved conscious perception when no alerting tone was presented. Nevertheless, given the low temporal resolution of fMRI these results should be taken with caution, and could be nicely complemented in future studies using EEG techniques and intra-cranial human recordings.

Functional connectivity analyses demonstrated that ACC-caudate coactivation was related to seen reports, while ACC-occipital cortex functional coupling was related to unseen reports. In another study^41^, ACC activation was observed in participants who were aware of, and capable of reporting, the changing of cue predictiveness when performing a Posner-type task, as compared to other participants who were unaware of the cue-target contingencies. Altogether, this evidence is consistent with the proposed role of ACC in purposeful behaviour and in the monitoring of its consequences. Consistent with this hypothesis, the ACC, together with the right middle frontal gyrus, has been associated with judgments of confidence in perceptual decision making^42^. Functional connectivity between the right middle frontal gyrus and both the contralateral prefrontal cortex and the visual cortex increased during metacognitive reports. Moreover, activation of the ACC, right posterior parietal cortex, and bilateral middle frontal gyrus correlated negatively with confidence reports. These brain activations are similar to those observed in our study. However, activations in the Fleming *et al*.’s study were related to post-decisional stages of processing, while in our study, brain activations were analyzed during the orienting period, and therefore, before the relevant target or the response occurred. Future research using techniques with better time resolution such as electroencephalography or transcranial magnetic stimulation could further demonstrate the time course of the contribution of the frontal network including the ACC, inferior and middle frontal lobes, in perceptual decision making.

Methodologically, it is important to note that in studies investigating the neural correlates of consciousness, researchers usually focus on brain activations related to seen and unseen reports, and therefore, having a manipulation in which target contrast is different for each of the manipulated conditions can be a methodological confound. In those studies, it might be difficult to disentangle the neural correlates of consciousness from the brain activations elicited by perceptually different stimuli. However, in the present paper, regional activation and functional connectivity analyses were restricted to the cue period, and therefore, the fact that target contrast is different for each experimental condition is not confounded with the effects of conscious perception *per se*, because the target related activations are not included in the time-period here examined. Nevertheless, in order to better understand the effect of target contrast in our results, we repeated all the ANOVAs for the parameter estimates, time-course, and pairwise functional connectivity analyses including target contrast for tone trials minus target contrast for no tone trials as a covariate. If target contrast were an important confounding factor in the data reported in the present manuscript, then the Alerting State x Awareness interactions should be modulated by this covariate. However, results demonstrated that none but one of the reported interactions in the parameter estimates, time-course, and pairwise functional connectivity analyses were significantly modulated by the covariate (all ps > 0.225). The only exception was the Alerting State x Seen x Time interaction observed in the thalamus, F(8,120) = 3.96, p < 0.001, which was significantly modulated by the covariate, F(8,120) = 2.07, p = 0.043. Results demonstrated a larger interaction for participants having smaller (rather than larger) differences in contrast for tone-present as compared with tone-absent trials. Similarly, we repeated the ANOVA for the time-course analysis, which showed a significant interaction between Validity and Awareness. Again, this interaction was not modulated by another covariate (target contrast for valid trials minus target contrast for invalid trials), F < 1. Therefore, our results clearly demonstrate that differences in target contrast were not a crucial confounding factor in our research, possibly because our analyses were related to the cue-period rather than to the target period.

Another methodological consideration is related to the fact that using near-threshold stimulation, accuracy to respond to the orientation of the Gabor lines was higher for seen as compared to unseen targets (accuracy was at chance levels for the latter). Some important concerns have started to emerge, because when studying the neural correlates of consciousness, objective performance can be confounded with consciousness^43^,^44^. It is important to notice, however, that in ecologically valid situations, we frequently do not respond to information that is not consciously reported. Therefore, although it is theoretically important to study conditions in which stimuli differ in their conscious access but not in objective performance, there are many real-life situations in which we correctly discriminate seen stimuli, but we respond at chance (or do not respond at all) to unseen stimuli. Moreover, in the present study we have made an effort in trying to equate seen and unseen conditions by asking participants to respond to all trials, even if the stimulus was not consciously seen^20^. Therefore, seen and unseen reports were equated in motor output, although motor preparation for seen stimuli might have differed from motor preparation when no stimulus was perceived. More work is needed to override this objective performance confound when exploring the neural correlates of conscious perception^43^,^44^.

Regarding top-down or endogenous attention, and in agreement with previous studies^18^,^21^, neither occipital nor fronto-parietal regions supported neural interactions between this attentional system and consciousness. The only significant interaction between endogenous spatial attention and conscious perception was observed in the thalamus, which demonstrated increased activations for seen as compared to unseen targets on invalid trials. This result observed with endogenous or top-down spatial orienting contrasts with the large fronto-parietal network supporting the interaction between exogenous attention and conscious perception^20^, which is consistent with the theoretical claims proposing the behavioural and anatomical dissociation between exogenous and endogenous spatial orienting^45^,^46^,^47^.

Many studies have reported dissociations between endogenous (or top-down) attention and conscious perception^48^. However, while a series of studies have demonstrated that endogenous attention does not increase perceptual sensitivity to detect near-threshold targets (see e.g. ref. 26), endogenous attention can modulate other measures of consciousness that are more bias-prone, such as the percentage of seen targets^49^, or target contrast to report a given percentage of the targets^18^. Although activations in fronto-parietal networks, typically associated with attentional orienting, are similar for consciously reported and unreported near-threshold targets, subcortical regions related to spatial orienting do show an interaction between endogenous attentional orienting and conscious perception. Both MEG and EEG techniques are less appropriate to measure subcortical activations, which might explain the absence of interactions between endogenous orienting and conscious perception observed in previous studies^18^,^21^. Moreover, Wyart and Tallon-Baudry’s MEG study focused on the interactions between spatial attention and consciousness within the occipital cortex, and was thus insensitive to the thalamic interaction that we observed in the present study.

Our results add to the large literature suggesting that the thalamus may serve a general purpose function supporting large-scale cerebral dynamics associated with goal-directed behaviors and consciousness^34^, and are in line with neuropsychological studies demonstrating that neglect can occur after lesions involving the thalamus^50^,^51^,^52^. The parietal cortex is interconnected with the dorsal pulvinar nucleus of the thalamus, and accordingly, inactivation (in monkeys^53^,^54^) or damage (in neglect patients^55^,^56^) of the dorsal pulvinar produces deficits in orienting attention to the contralateral space.

These observations are also consistent with previous models and empirical data emphasizing the importance of thalamo-cortical loops in conscious perception^57^,^58^,^59^,^60^. Tononi and Edelman^57^,^61^ proposed that the synchronized activity of large population of thalamo-cortical neurons is associated to conscious perception. Other authors suggested an even more important role of the thalamus, indicating that the synchronized activity of neuron dendrites within the dorsal thalamic nuclei, supports conscious perception^62^. The role of the thalamus in consciousness is also well established in studies demonstrating that lesions of this region produce alterations in the state of consciousness^34^,^63^,^64^,^65^.

To conclude, the present study demonstrated brain interactions between spatial attention and conscious perception in the thalamus, and between phasic alerting and conscious perception in a midbrain-thalamic-anterior cingulate cortex circuit. Coactivations between the ACC and caudate nucleus were demonstrated as a key mechanism determining conscious perception. Large interactions between phasic alerting and conscious perception were demonstrated in critical regions previously related to the alerting attentional system. The present results add to our understanding of how different attentional systems improve conscious perception and how these interactions are implemented in the human brain.

## Methods

### Participants

Nineteen right-handed voluntaries took part in the experiment (10 females, mean age 26 years, SD = 4). Additional data from one participant were excluded from the analyses due to excessive head motion during imaging. All participants had no neurological or psychiatric conditions and followed the safety requirements to underwent MRI scanning. They were naive to the purpose of the experiment, had normal or corrected-to-normal vision, and received monetary compensation for their participation. They gave signed informed consent to participate in the experiment. The study was reviewed by the INSERM ethical committee and received the approval of an Institutional Review Board (CPP *Ile de France* 1, Paris, France). The study was carried out in accordance with the approved guidelines.

### Stimuli

E-prime software was used to control the presentation of stimuli, timing operations, and behavioral data collection. Images were projected to the head of the bore of the scanner via a display projector (Epson EMP-8300, 1024 × 768, 60 Hz) and viewed with a mirror attached to the head coil. Three black boxes (6° height × 5.5° width) were displayed, one in the center of the display, and the other two centered 8.5° to the left and right. The fixation point consisted of a black plus sign (0.5° × 0.5°) presented on the central box. The spatial cue consisted of an either a blue or yellow color circle subtending 0.5° in diameter, presented at the fixation point. This spatial cue was either blue or yellow, indicating that the targets will likely appear on one side or the other (with color left and right side assignments being counterbalanced between participants). We used color instead of arrows as cues to be sure to induce a purely endogenous orienting of attention. White noise (22.050 Hz, 74 dB) was presented through the headphones as the alerting cue. A custom Matlab script was used to create 100 Gabor stimuli (4 cycles/deg. spatial frequency, 3° in diameter, SD of 0.3°), with a maximum and minimum Michelson contrast of 0.92 and 0.02, respectively.

The target contrast was adjusted during the fMRI session, so that the percentage of consciously perceived targets was ~50%. This titration procedure was done based on individuals’ performance on the practice task (see task description below), and carried out independently for valid, invalid, tone present, and tone absent trials. All participants started with a high contrast stimulus (Michelson contrast = 0.184), which was well above the threshold of conscious perception. Every 40 trials, target contrast was automatically adjusted using a “one-up-one-down” procedure, until participants perceived ~50% of targets for each condition (valid, invalid, tone present, and tone absent trials) in at least two consecutive blocks of 40 trials. If the percentage of correct detection rates was above 55% of the trials, Gabors at the immediately following lower contrast level (Michelson contrast minus 0.009) were used for the next block. Inversely, if the percentage of correct detection rates was below 45% of the trials, Gabors at the immediately following higher contrast level (Michelson contrast plus 0.009) were used for the next block. Accuracy of the objective response was similarly titrated so that correct discrimination performance was between 65% and 85% (Gabor grating tilt orientation ranging from 1° to 10°, which tilt orientation changing 1° if accuracy was larger than 65% or lower than 85% every 40 trials). The experimental session started when participants felt comfortable with the task, and performance converged at a target contrast yielding ~50% seen targets for each condition (valid, invalid, tone present, and tone absent trials). This titration procedure continued during the whole experiment (this time adjusting target contrast every 46 trials: 40 + 6 catch trials) to prevent factors such as practice or fatigue from influencing conscious perception.

### Procedure

Cue color predicted the spatial location of the target on 70% of the target-present trials. Participants were informed about the predictive value of the cue (see Fig. 1). Although they were not told the exact amount of trials in which the cue predicted the target’s location, they were encouraged to take this information into account in order to respond more accurately. The alerting cue was presented on 50% of the trials.

Participants were asked to provide two responses to each target consecutively, by making key presses on a 2-horizontally-aligned-button fiber-optic box. First, they were required to discriminate the orientation of the Gabor (objective task) by pressing, with their right hand, a left situated key if the target was oriented to the left, and a right situated key if the target was oriented to the right. Participants were encouraged to respond to every trial as fast and as accurately as possible. Even if they did not see the stimulus, they were encouraged to guess the correct response [this response mapping could cause Simon-like compatibility effects; faster responses when responding with the same hand where the target was located than with the opposite hand. However, the percentage of seen targets was not modulated by this Simon-like effect (50% seen targets for compatible trials, and 48% for incompatible trials, p = 0.107)].

Second, participants had to report if they consciously detected the appearance of the Gabor (subjective task) as accurately as possible. This time, we encouraged participants to take their time to respond correctly. We presented participants with two arrow-like stimuli, one below and the other one above the fixation point (>>> or <<<). The vertical arrangement of the arrow-like stimuli ensured that participants could not prepare a lateralized response in advance, associated with the location of the Gabor. We provided participants with 3 vertically aligned keys (to-be-pressed using the left hand). The upper key always corresponded to the arrow presented above the fixation point; the middle key was associated with the arrow presented below the fixation point; and, the lower key was used to indicate that the Gabor was not seen. No target was presented on 13% of the trials. In target-absent trials, participants were also required to give the objective response, and then report whether they saw the target or not. Trials could finish with a further fixation period (jitter fixation) (see Fig. 1).

The experiment consisted of two sessions with 5 functional scans each. Each functional scan lasted 12 min. Across both sessions, participants encountered a total of 920 trials (120 of them were target-absent trials). Valid, invalid, tone, no tone, and target-absent trials were presented in a pseudorandomized order during scanning. Valid trials accounted for 70% of the target-present trials. The jitter fixation and the order of trial types within each scan was determined with an optimal sequencing program designed to maximize the efficiency of recovery of the Blood-Oxygen-Level Dependent (BOLD) response Optseq II. The jitter fixation periods were interleaved with the experimental trials as determined by the optimization program.

### fMRI data acquisition

Whole-brain fMRI was conducted on a 3-T Siemens TRIO whole-body MRI scanner at the CENIR MRI center (Salpêtrière Hospital in Paris) using a standard whole-head coil. Functional images were acquired using a gradient-echo echo-planar pulse sequence (TR = 2000 ms, TE = 25 ms, 39 contiguous 3-mm cubic axial slides, no inter-slice gap, flip angle = 75°, field of view = 220 mm, 372 volumes acquired per run). Prior to each functional scan, four volumes were discarded to allow for *T*_*1*_-equilibration effects. High-resolution T1-weighted anatomical images were also collected.

### fMRI data analysis

Standard preprocessing routines and analyses were conducted in SPM8 (Welcome Department of Cognitive Neurology, London). Images were corrected for differences in timing of slice acquisition and were realigned to the first volume by means of rigid-body transformation. Then, functional images were spatially smoothed using a 4-mm full width at half-maximum (FWHM) isotropic Gaussian kernel. Next, motion parameters obtained from realignment were used to inform a volume repair procedure (ArtRepair; Stanford Psychiatric Neuroimaging Laboratory) that identified bad volumes on the basis of within-scan movement and signal fluctuations, and then corrected bad signal values via interpolation. A volume-by-volume correction with a 1.5 mm threshold was applied, which did not remove more than 15% of the volumes in any participant of the final study sample. After volume repair, structural and functional volumes were corregistered and spatially normalized to T1 and echo-planar imaging templates, respectively. The normalization algorithm used a 12-parameter affine transformation together with a non-linear transformation involving cosine basis functions. During normalization, the volumes were sampled to 3-mm cubic voxels. Templates were based on the MNI305 stereotaxic space. Then, functional volumes were spatially smoothed with a 7-mm FWHM isotropic Gaussian kernel. Finally, time series were temporally filtered to eliminate contamination from slow drift of signals (high-pass filter: 128 sec).

Statistical analyses were performed on individual participants’ data using the general linear model (GLM). fMRI time series data were modeled by a series of events convolved with a canonical hemodynamic response function (HRF). Two different fMRI GLMs were used in the analyses. First, we used an event-related model where the three phases of each fMRI trial (i.e., Cue, Target/Objective Task, Subjective Task) were modeled separately as events, time-locked to their onset time. This model was specifically intended to examine neural changes restricted to the cue-period and used in whole-brain contrast, region-of-interest (ROIs) and functional connectivity analyses (see below). This model included a total of 11 regressors of interest: cue valid seen, cue valid unseen, cue invalid seen, cue invalid unseen, cue tone present seen, cue tone present unseen, cue tone absent seen, cue tone absent unseen, cue for target absent trials, target/objective task, and response/subjective task. Valid, invalid, tone present, and tone absent fMRI trials were sorted as ‘seen’ or ‘unseen’ (i.e., Awareness) according to participants’ responses on the subjective task. The cue period for target absent trials, the target/objective task, and the response/subjective task were modeled separately, but were excluded from the main analyses.

Of importance for this first GLM, since cue and target periods followed each other in close succession in all trials it was relevant to examine to what extent the activation observed during the cue period could be influenced by target processing-related activation. To examine this possibility, we performed voxel-wise and ROI based comparisons involving the cue period from target absent trials and the cue period for target present unseen trials. These comparisons did not yield any significant effects in a whole-brain F contrast among these conditions (p < 0.001 uncorrected, voxel-level threshold) and simple-effects analyses comparing the parameter estimates for these two conditions in all the ROIs featured in the present work (all ps ≥ 0.278; see below for a detailed description of ROI analyses).

Second, we modeled each fMRI trial as a 6 s period, time-locked to the onset of the cue presentation. This model was intended to examine time-course analysis in regard to fMRI trials. This model included the same regressors of interest described for the first GLM, except for the target/objective task and the response/subjective task (i.e., total of 9 regressors). The motion parameters for translation (x, y, z) and rotation (yaw, pitch, roll) were also included as covariates of noninterest in these GLMs. Also, for both GLMs, conditions were convolved with a HRF function in SPM8. The resulting functions were used as covariates in the GLMs, along with a basic set of cosine functions and a covariate for session effects. The least-squares parameter estimates of the height of the best-fitting canonical HRF for each condition were used in pairwise contrasts.

Contrast images, computed on a participant-by-participant basis were submitted to group analyses. At the group level, whole-brain contrasts between conditions were computed by performing one-sample *t* tests on these images, treating participants as a random effect. Whole-brain maps involving all participants were thresholded at p < 0.05 cluster-level FWE rate correction for multiple comparisons (with a voxel-level inclusion threshold of p < 0.001), for the consciously seen targets versus unseen targets (Seen > Unseen) contrast (see Table 1). These whole-brain maps were restricted to the cue period (i.e., 700 ms fixation +300 ms spatial cue +600 ms fixation + 32 ms alerting cue + 368 ms fixation).

ROI analyses, also restricted to the cue period, were performed with the MARSBAR toolbox. ROIs consisted of significantly active voxels for clusters identified from the Seen > Unseen whole-brain comparison across all participants within specific MARSBAR anatomical ROIs. From this contrast, we only explored ROIs previously implicated in attentional orienting (IPL, SPL, and FEFs) and alerting processes (ACC, SMA, caudate, and thalamus) (see *Introduction*). The center of mass of each ROI is reported in Fig. 3.

We also performed time-course analyses for the fMRI trials. BOLD activity time series, averaged across all voxels in each ROI, were extracted for each functional run. Mean time courses for each trial were then constructed by averaging together appropriate trial time courses, which were defined as 12-secs windows of activity after trial onset. These condition-averaged time courses were then averaged across functional sessions and across participants.

Finally, we assessed functional connectivity *via* the beta correlation method, implemented in SPM8 with custom Matlab scripts. These analyses were restricted to the cue period. The canonical HRF in SPM was fit to each occurrence of each condition and the resulting parameter estimates (i.e., beta values) were sorted according to the study conditions of interest (endogenous orienting -valid and invalid, alerting state -alert and non-alert, and awareness -seen and unseen) to produce a condition-specific beta series for each voxel. Two different functional connectivity analyses were performed: (1) pairwise connectivity between the ROIs extracted from the Seen versus Unseen contrast (IPL, SPL, FEFs, ACC, SMA, caudate, and thalamus); and (2) whole-brain functional connectivity with the right ACC as the seed region.

First, for the pairwise functional connectivity analyses we calculated beta-series correlation values for each pair of ROIs and participant. Since the correlation coefficient is inherently restricted to range from −1 to +1, an arc-hyperbolic tangent transform was applied to these beta-series correlation values (r values) to make its null hypothesis sampling distribution approach that of the normal distribution. Then, these Fisher’s z normally distributed values were submitted to repeated-measures ANOVAs with the factors of Validity, Alerting State, and Awareness. To control that differences in coupling strength were not determined by differences in the cluster size of the functionally defined ROIs, we used 5-mm radius spheres centered at the highest local maxima of each ROI.

Because the right ACC was the region demonstrating larger interactions in the previous pairwise functional connectivity analysis, for the whole-brain functional connectivity analysis, the beta series associated with this region were correlated with voxels across the entire brain to produce beta-correlation images. Contrasts between beta-correlation images were also subjected to an arc-hyperbolic tangent transform to allow for statistical inference based on temporally coupled fluctuations with this region. Seen > Null and Unseen > Null t-tests were performed on the resulting subject contrast images to produce group correlation contrast maps with a threshold of p < 0.01, FWE voxel-level corrected for multiple comparisons.

## Additional Information

**How to cite this article**: Chica, A. B. *et al*. Interactions between phasic alerting and consciousness in the fronto-striatal network. *Sci. Rep.* **6**, 31868; doi: 10.1038/srep31868 (2016).

## Figures and Tables

**Table 1:** Activations for consciously seen versus unseen targets (Seen > Unseen) whole-brain contrast thresholded at p < 0.05, FWE cluster-level corrected.

Brain area	MNI (*x, y, z*)			z-score	voxels	BA
Frontal						
L SMA	−3	9	51	5.52	227	32/6
	−3	21	48	4.69		
L ACC	−3	9	30	4.37	228	24
R IFG	48	9	27	3.82	118	9
R FEF	27	−3	57	3.61	131	6
Parietal						
L Inferior Parietal	−36	−42	42	4.61	178	40
	−27	−72	30	4.23	134	7
L Superior Parietal	−30	−69	54	3.63	137	7
Subcortical						
Thalamus	−9	−21	3	6.09	819	
(including putamen and	−3	−27	−3	5.01		
caudate nuclei)	−9	−18	−9	4.96		

**Table 2:** Significant Alerting State x Awareness interactions in the Parameter Estimate analysis.

Brain Region	F	MSE	p	η_p_^2^
ACC	5.91	0.207	0.027	0.269
Caudate	5.841	0.129	0.028	0.267
FEF	5.161	0.314	0.037	0.244
SMA	5.907	0.478	0.027	0.269

All regions demonstrated larger activation for seen as compared to unseen reports, especially for no tone trials (see Fig. 2).

**Table 3:** Statistically significant interactions between Alerting State, Awareness, and Time in the time-course analysis.

Brain Region	F	MSE	p	η_p_^2^
ACC	3.311	0.021	0.009	0.171
Caudate	2.431	0.009	0.042	0.132
FEF	3.825	0.020	0.004	0.193
Thalamus	2.568	0.007	0.033	0.138
SMA	4.436	0.027	0.001	0.217

All regions demonstrated larger activation for seen as compared to unseen targets, especially for no tone trials, although differences started at different time points (see Fig. 4).

**Table 4:** Significant interactions between Alerting State and Awareness in the pairwise functional connectivity analyses with a seed placed in the right ACC.

Brain Region	F	MSE	p	η_p_^2^
Caudate (right)	4.958	0.072	0.039	0.216
FEF	7.879	0.351	0.012	0.304
SMA	5.922	0.139	0.025	0.247
